# The evolution of language by sexual selection

**DOI:** 10.3389/fpsyg.2022.1060510

**Published:** 2022-12-22

**Authors:** Robert Worden

**Affiliations:** Wellcome Centre for Human Neuroimaging, Institute of Neurology, University College London, London, United Kingdom

**Keywords:** language, sexual selection, theory of mind, social intelligence, pragmatics, relevance, cognitive linguistics, speed limit for evolution

## Abstract

Most accounts of the evolution of language assume that language and greater intelligence are beneficial adaptations, leading to increases in survival fitness. These accounts emphasise natural selection, with language as an adaptation to the habitat, placing less emphasis on sexual selection and reproductive fitness. An account of language evolution by natural selection alone faces problems in accounting for the prodigious power and expressivity of human language. Modern language (and its recent antecedents) would appear to offer only small incremental benefits over simpler language, which would require a smaller brain with smaller metabolic costs. Accounts by natural selection also face problems in accounting for the uniqueness of human language and intelligence. I therefore consider a hybrid account, in which both natural selection and sexual selection played a role in the evolution of language and intelligence, probably at different times. Specifically, in this account, early language was driven by natural selection to collaborate. Then later humans became subject to sexual selection for superior intelligence, with language acting as the main display mechanism for intelligence. It is hard to determine the relative roles of natural and sexual selection over the time course of the evolution of language. In the later stages, sexual selection to display intelligence drove a runaway selection process towards powerful modern language. This hybrid account retains the benefits of accounts by natural selection, while also accounting for the prodigious power of human language and intelligence, and for its uniqueness compared to other primates. Sexual selection often leads to traits which are unique to a species, and are exaggerated beyond natural needs. On this account, the capability for language may have evolved in the order: (1) pragmatics and a theory of mind; (2) using single words and constructions; (3) learning and using syntax. In this model, relevance-based pragmatics evolved before language; then, single words and constructions came into use; and later, syntax condensed out of pragmatics, as a codification of some pragmatic rules of inference. This order requires only incremental extensions of primate cognition, and agrees with the order in which children learn language.

## Introduction

It is widely assumed that language, by enabling us to think rationally and to cooperate with other people, increases the fitness of mankind compared to other primates. For instance, Feldman (2008) writes: *“Everyone agrees that expressive language conveys very significant evolutionary advantages for groups that can use it*.”

This consensus sits awkwardly with four facts, which are not readily consistent with the picture of language as a selective adaptation to our habitat:

- For most of the time in which we have had the capacity for language, it has not led to increased fitness, as measured by human population size. For most of this period (of 2 million years) mankind has been a marginal species in Africa – with an effective breeding population of the order of 10,000, less than chimpanzees. This suggests that for some extended periods while language was evolving, it was not a source of major fitness benefits.
- Our greater intelligence and capacity for language come with large costs. The most important cost is the huge metabolic requirement of our large brains. Any account of the survival benefits of language needs to gives enough benefit at any time to offset the increase in food requirements from the size of the human brain at the same time.
- If language conferred survival benefits in the wild, most of its benefits could come from a communication capability which was much slower and less powerful than modern human language, enabling us to communicate ideas over minutes or hours—not within seconds, as we do. Modern language is highly over-engineered for any habitat-related function; implying that it must also have been over-engineered for the later parts of its evolution.
- If language had been an evolutionary response to the demands of the habitat, then other ape species could have evolved similar responses to similar selection pressures; yet they have hardly evolved in the direction of language and cooperation.

This evidence—particularly the last two points—does not sit easily with an account of language as a selective adaptation to habitat-related selection pressures. Together, these points pose a hard question: why did language evolve?

This paper explores a possible answer to the question. Language has evolved in part by natural selection, and in part by sexual selection to display superior intelligence. Sexual selection is ubiquitous; it is well understood, and it accounts well for many unique features of human language. As described in the paper, a hybrid model of natural and sexual selection is consistent with the four facts above.

The paper is organised into four parts:

- In section “Four difficulties for an account of language as the result of natural selection,” the four difficulties above, for an account of language arising solely through natural selection, are described in more detail.
- Section “Outline of the course of language evolution” outlines the course of language evolution that is proposed in this paper, through a combination of natural and sexual selection.
- Sections “The theory of sexual selection” and “Intelligence and language evolved by sexual selection” describe the theory of sexual selection, and describe how language evolved by sexual selection, and how sexual selection explains the over-engineered speed and expressive power of modern language.
- In sections “Stages in the evolution of language,” “Three phases in the evolution of language,” “Evolution of the brain,” “Comparison of scenarios for the evolution of language,” and “Other accounts of human brain enlargement” some consequences of the proposed origin of language are explored.

Section “Stages in the evolution of language” describes a possible order in which our language capability may have evolved, in a pragmatics-first order. Section “Pragmatics before language” describes how the primary skill required to display intelligence is pragmatics (Levinson, 1983; Sperber and Wilson, 1986; Grice, 1989), which depends on the ability to build up a shared understanding with a conversational partner, over several turns in an extended conversation. It also requires an enhanced Theory of Mind (ToM) capability. Pragmatic skills may have evolved to a time before there was spoken language, when people used actions to convey information. Later, as in section “The emergence of symbols,” symbols (words and other constructions) emerged. Finally, grammar emerged, as in described section “Syntax emerged from pragmatics,” as a streamlining of pragmatic exchanges for faster information transfer, driven by sexual selection. Section “Neurobiological foundations for language” addresses the neural and physical adaptations necessary to support this course of evolution.

Section “Three phases in the evolution of language” describes the three-stage picture of language evolution which emerges from this account. Section “Evolution of the brain” discusses some other issues in the course of the evolution of the brain. Section “Comparison of scenarios for the evolution of language” compares this account with some alternative scenarios for the evolution of language, using the criteria of Számadó and Szathmáry (2006) and Botha (2016). Section “Other accounts of human brain enlargement” addresses some alternative accounts of the causes of human brain enlargement.

## Four difficulties for an account of language as the result of natural selection

There are two main forces driving evolution: natural selection (the need to survive to adulthood) and sexual selection (the need to reproduce). An account of language evolution by natural selection alone faces a number of difficulties (or equivalently, has some weaknesses), which are discussed here in sections “Language has not greatly increased survival” to “Natural selection struggles to account for the large differences between humans and other great apes.” These difficulties are not equally serious; for some of them, there are now, and there may be in future, evolutionary considerations which mitigate the problems. However, taken together, the difficulties provide sufficient motivation to explore an account in which language arose not only by natural selection, but by a hybrid of natural and sexual selection. This section is intended to provide motivation for the sections which follow; the strongest motivation is in section “Language is over-engineered.”

### Language has not greatly increased survival

The date of the origin of human language is not known. On the assumption that the descent of the human larynx evolved for language (Lieberman, 2003) the approximate date may be 200,000–300,000 years ago. This assumption is controversial (Fitch, 2000) and should be seen as a lower limit, because language could previously have used modalities other than speech, or started without modern human vocal capabilities.

Language requires intelligence, which requires a larger brain. From the fossil record, the enlargement of our brains compared with the brains of other great apes dates back more than 2 million years. An important milestone may have been the invention of cooking about 1-2 million years ago (Wrangham, 2009)—enabling us to digest more food, to support a further enlarged brain.

Conservatively, therefore, the evolutionary origin of the human language capability dates to something between 200,000 and 2 million years ago (the range of possible dates is revisited in section “Three phases in the evolution of language” of the paper).

It is commonly believed that the capability for language is wholly beneficial, and has increased our ability to survive. However, that view is not consistent with human populations over the time period. Had language conferred a large survival benefit (which caused language to evolve), then around the time of the evolution of language we would expect to find a significant increase in human population, resulting from the increased survival.

It is now possible to make approximate estimates of human population size over the whole Pleistocene era (from about 2.5 million years ago to 11,000 years ago) from modern human genetic data. Harpending et al. (1998) write: “*Several nuclear loci are informative about our ancestral population size during nearly the whole Pleistocene. They indicate a small effective size, on the order of 10,000 breeding individuals, throughout this time period*.” A more recent study (Huff et al., 2010) gives a similar figure at a date 1 million years ago. Genetic bottlenecks in mitochondria and Y chromosomes further constrain any estimate of population size at the time of “Mitochondrial Eve” and “Y Chromosome Adam” in the 300,000–500,000 year range. Harpending et al. make several independent estimates of population size over this time range.

So if the date of evolution of our language capacity was at least 200,000 years ago, for at least 95% of the period when we had language (or 99.5% of the longer period when we have had enlarged brains) mankind has not been a particularly successful species—limited around 10,000 breeding individuals, typically a smaller population than chimps. Only in the last 10,000 years, since the emergence of agriculture, has mankind prospered in terms of larger populations. This shows that language in itself has not been a driver of increased survival. The greater fitness and increased population of mankind came with civilization, which started long after the capacity for language had evolved, at around 10,000 BC.

In response to this difficulty for the account of language evolution by natural selection, a possible response is that increased survival does not always result in an increased population—for instance, if new traits restrict a species to some narrow ecological niche. This response has some force, but it does not entirely remove the difficulty. The simplest expectation from any increase in survival is that it will lead to increased occupation of some niche, or to an ability to inhabit more niches; this is usually what occurs in a “successful” species. Increased survival without increased population requires some special pleading; and occupying a smaller niche clearly increases the risk of extinction.

### Language has large costs in survival fitness, and may not repay those costs

**Figure 1:**
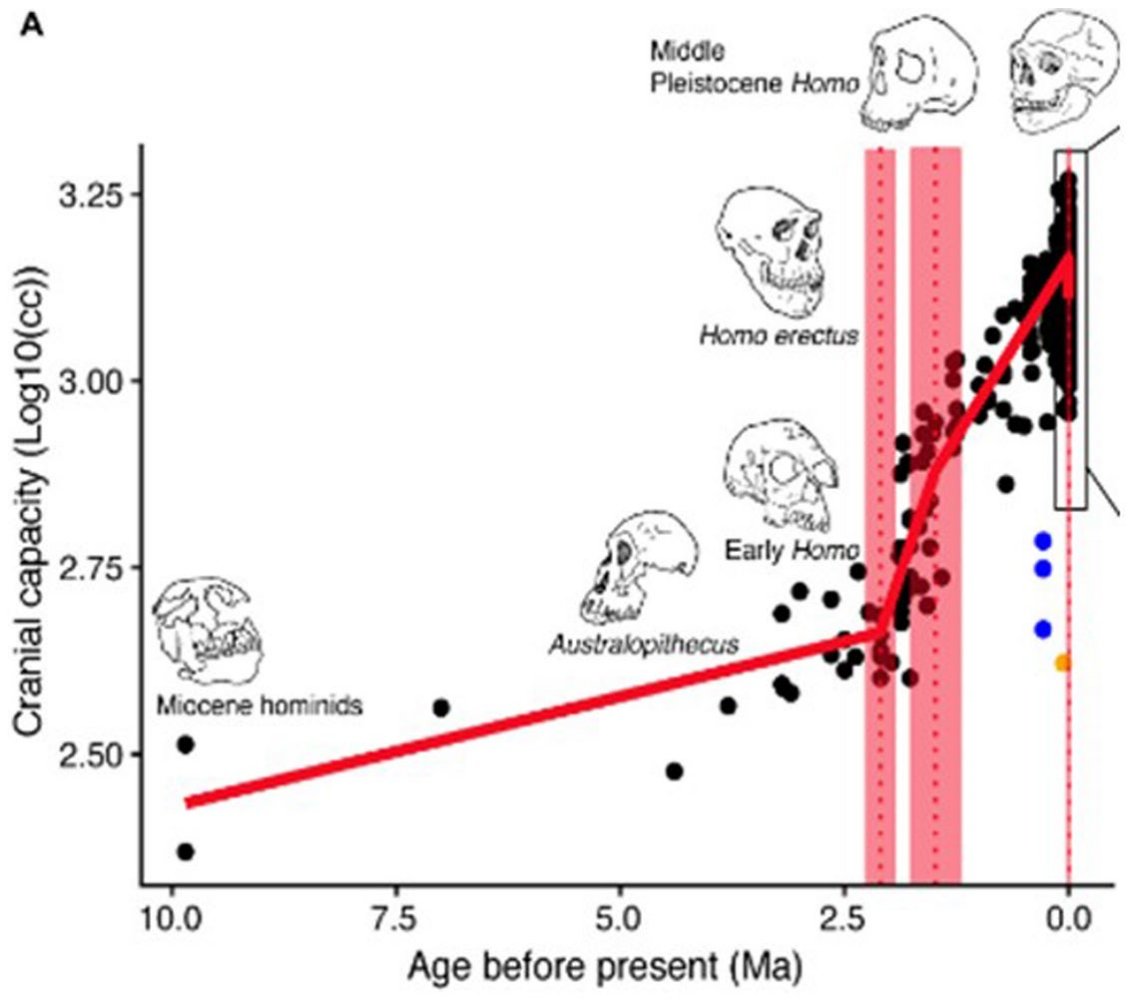
Evolution of human brain size over the past 10 million years, from DeSilva et al., 2021.

Our brains have very large energy requirements, which are estimated to be approximately 20% of our total metabolic requirements. 20% of the food we eat is needed just to feed our brains (Raichle and Gusnard, 2002). As the diagram below (DeSilva et al., 2021) implies, about half of this extra food requirement – or 10% of our total food requirement—has arisen during the last million years (see Figure 1).

Language requires intelligence, and so requires a larger brain. For this, we need to be better at gathering food. Great apes and hunter-gatherers spend a large part of their day gathering food—so it is reasonable to suppose that gathering food is a limiting factor for their survival fitness.

- One account is that cooperation and language have enabled us to be better (Tomasello, 2014) hunters and gatherers – but is an increase in food gathering efficiency as large as 10%–20% plausible? This is not an easy question to answer, for several reasons: mainly, it does not pertain to the full extra metabolic costs of a modern human brain, but to some smaller incremental costs associated with an earlier stage of language evolution.
- Chimpanzees can collaborate in hunting without language, albeit selfishly and not easily (Boesch, 2002).
- Foraging is a largely solitary activity, not greatly enhanced by cooperation. The knowledge required to forage can be spread by imitation and following, as other primates do.
- The combination of collaborative hunting and weapons has greatly increased the efficiency of human hunting in recent evolutionary times, but it is hard to say how much this is relevant to the early development of language.

So it remains possible that the use of early language in planning hunting (Számadó, 2010) or tool-making, or both, gave sufficient benefit to offset the metabolic costs of a larger brain. However, as discussed below in section “Language is over-engineered,” at later stages, more sophisticated and faster language may have given diminishing returns in this respect—requiring something else to drive the later stages of language evolution.

Other accounts have been suggested, and are surveyed in Számadó and Szathmáry (2006). For instance, Dunbar (1993) proposes that language evolved as a more efficient form of grooming—allowing group sizes to increase from under 50 (other primates) to over 150 (humans). It is hard to see how an increase in group size can increase the efficiency of food gathering, by 10% or more. Rather, bigger group size is expected to make food gathering less efficient, requiring more travel, because a larger group needs to forage or hunt over a larger area.

Similarly Deacon (1997) proposes that language enables the agreement and checking of social contracts within a group. It is hard to see how this on its own would have led to much more efficient food gathering.

So it is possible that cooperation and early language have increased our effectiveness at food gathering by as much as the amount required to compensate for their extra metabolic costs. However, the greatest difficulty for an account by natural selection is the next one—that even if early language could increase our survival fitness in the wild, we would not need the fast, highly expressive language that we now have, to do so.

### Language is over-engineered

Human language is a remarkable communication system. As far as we know, no other species has anything like it. Some of its outstanding features are:

- We have huge vocabularies, up to 50,000 words.
- There is almost no limit to the range of topics we can talk about.
- We can communicate and understand complex ideas in seconds.
- We can pack almost unlimited meanings into a single sentence, using complex nested syntax.
- We learn language in childhood, without effort or coaching.
- We convey meanings very rapidly using linguistic short-cuts such as pronouns or ambiguous noun phrases.
- We rapidly infer one another’s conversational intent, and take our conversational turns appropriately within an average of 200 milliseconds (Levinson and Torreira, 2015).

In these respects, present-day language is much faster and more powerful than would be needed to serve any purpose related to survival in our natural habitat. This high level of over-engineering at the present day implies that even in the past, over much of the period when language ability was evolving, our language capability has been to some extent over-engineered.

If language had evolved to help us in hunting, or gathering food, or finding shelter or caring for loved ones, or living in larger groups—all of which happen over timescales of minutes or longer—then something much slower and less powerful than modern human language could have sufficed. We could speak in slow, simple sentences, using vocabularies of only a few hundred words, taking minutes to express any meaning. We would not need all the short-cuts, speed, and expressive power of modern language. The benefits in fitness of these extra abilities are marginal. There is little benefit in shaving seconds off information exchanges which would serve their purpose even if they took many minutes. This means that the incremental benefits of greater speed, as language evolved to its modern form, were also marginal.

Great apes and hunter-gatherers spend many hours of their waking day finding food. If cooperation and language could make this activity more efficient by as much as a few percent, then there are fairly long times available, of minutes or hours, for it to do this. There would be no need to pack complex meanings into a few words or into single sentences; or to take turns in conversational exchanges within 200 ms, as we do. The prodigious power and speed of modern human language is not driven by the need to find food.

Put another way, the fitness benefits of supercharged language are subject to sharply diminishing returns, approaching a ceiling as the power and speed of language increases. In a natural habitat, there are only limited numbers of food sources available, no matter how fast or eloquently you talk about them. That places a limit on how much a faster language ability can benefit you. The probability of finding enough food for the next few days can be raised by some amount through exchanging information, and no further—using slow language or fast language.

Other proposed uses of language do not require great speed. Gossip and grooming (Dunbar, 1993) do not require haste; if anything, they require extended times for emotional bonding. Similarly, important social commitments (Deacon, 1997) do not need to be made in seconds.

From another viewpoint, during our hunter-gatherer past, the scope of language seems to have grown far beyond its practical utility. Had language evolved to help us hunt, or gather food, or escape predators, it might have evolved with special-purpose constructs for those purposes, along the lines of vervet monkeys’ alarm calls (Cheney and Seyfarth, 1990). This would have required much less brainpower than the general-purpose language which we now have. This is another indication that general-purpose language is a display of intelligence, rather than an adaptation for survival.

One aspect of the power of modern language is our very large vocabularies or 60,000 words or more. Miller (2001, p. 369 onwards) has argued that these large vocabularies arose by sexual selection. Briscoe (2008) has argued against this, saying that “*To demonstrate that vocabulary size and distribution, or any other linguistic trait, is under sexual rather than natural selection, it is necessary to show that it would not evolve in any other way and does not contribute to communicative success. Miller fails on both counts*.”

Briscoe is not saying that the account from sexual selection is wrong; he is saying that it has not been demonstrated. In arguing this, Briscoe’s definition of sexual selection is narrow, essentially restricted to the use of language in “*courtship and seduction*”; whereas in this paper, powerful language can be used in any context, with any person, to display superior intelligence and so to gain status in the group leading to reproductive opportunities—as is the case for most primates. Briscoe’s argument is not relevant because he requires that large vocabularies should not “*contribute to communicative success*.” But as we will show later in the paper, communicative success in extended exchanges is a primary requirement for displaying intelligence. Large vocabulary, on any topic, is an indicator of intelligence.

### Natural selection struggles to account for the large differences between humans and other great apes

If natural selection has led to humans having greatly increased intelligence and language, there is a question as to why chimps and bonobos have not developed a similar capacity. There are three possible answers, none of which entirely remove the difficulty.

The first possible answer is that during the last 2 million years in which language has evolved, *homo* has been living mainly in a different habitat (savannah) from chimpanzees and bonobos (tropical rain forest; Bobe et al., 2002; Bobe and Behrensmeyer, 2004). Therefore, it could be said, different habitats have led to different selection pressures and to different levels of intelligence. However, the difficulty remains that the differences in intelligence and language are so extreme, that it seems hard to account for them without extreme differences in habitat challenges; and human intelligence seems to go far beyond the needs of a savannah habitat.

The second possible answer is that human language is such a prodigious mental ability that other great apes were not able to evolve it—only humans were capable of such a remarkable evolutionary “achievement.” However, this view is at odds with the known incremental nature of evolution, and the proven capability of evolution. It is similar to creationist arguments that evolving an eye is impossible.

Language had to start somehow—which must have been to make use of the cognitive abilities of the primate brain—from which it evolved incrementally. Then, if *homo* could evolve incrementally towards more powerful language, so could chimps; there cannot have been any fundamental block present in the chimp brain, but not in the pre-human brain—because at the start, they were the same brain.

A third possible answer is that mankind evolved to occupy a “cognitive niche” (Tooby, 1987), and that chimpanzees did not. The concept of a niche seems to introduce some new element into the discussion, but does it help in this case? A “niche” involves occupying some part of a habitat, and not occupying other parts. For instance, a cognitive niche might involve an ability to eat plants that have special defence mechanisms, by thinking of ways to counter their defences—and not eating other plants. However, humans and chimps are omnivores; neither species is a niche eater, dependent on a narrow range of foods; so a niche capability to eat certain plants would not count for much. Perhaps the main shortcoming of the cognitive niche theory as an account of language evolution is that whatever aspects of survival could be enhanced by sharing intelligent insights (food, predation, shelter and so on), it would not have required the fast, super-powerful language that we now have to do so; a much slower proto-language would have sufficed.

So while there are some possible accounts from natural selection of the large differences in intelligence and language between humans and other great apes, none of them are entirely satisfactory. In their evaluation of 11 different accounts of language evolution Számadó and Szathmáry (2006) found that no account gives a satisfactory answer to the question of human uniqueness.

If the accounts of language evolution from natural selection on its own face the difficulties (more or less serious) which have been described in this section, there are reasons to explore an account which does not rely on natural selection alone, but which also allows the possibility of sexual selection. That account follows.

## Outline of the course of language evolution

This section gives a summary of the course of evolution of the language capability which is proposed in this paper, in order to introduce and orient the detailed considerations of later sections.

The overall fitness of any animal is a product of its ability to survive to adulthood, and its ability then to reproduce and pass on its genes. This can be summarised as “Fitness = Survival x Reproduction.” Selection pressures can act on both terms of the product. The pressures to increase survival are called natural selection; the pressures on reproduction are called sexual selection. Sexual selection can affect any species at any time, and has an arbitrary, species-specific nature; it may be unrelated to habitat conditions, and once sexual selection for some trait has started, it typically continues rapidly in the same direction by a positive feedback process. This evolutionary positive feedback is the “runaway selection process” of Fisher (1915) and Fisher (1930). In order to be sexually selected, any trait must be visible to potential mates; it must be displayed.

Miller (2001) proposed that greater human intelligence and brain size evolved by sexual selection. This paper agrees with that account, and proposes that throughout most of the period of human brain enlargement (from about 2 million years ago) language has served as the principal means of display of superior intelligence to potential mates (and to other peers, to gain social status for reproductive purposes). This paper does not take Miller’s theory of sexual selection for intelligence as fact; it adopts it as a hypothesis, with the additional hypothesis that language has acted as the display mechanism for intelligence, to test what evidence they account for. As we will see, these hypotheses account for two facts which natural selection alone does not account for—the uniqueness of human intelligence, and the prodigious power of modern language.

Thus the key driver and cause of the evolution of our language capability, since about 2 million years ago, has been the need to display superior intelligence to our peers—because since that time, superior intelligence has been sexually attractive to humans.

We cannot know for certain why intelligence started to be attractive to potential mates—any more than we can know why certain patterns of bird plumage, or certain mating rituals, started to be attractive. One possible account is a serial hybrid model, in which habitat needs such as hunting or tool-making provided the initial impetus for early language; and then later, sexual selection to display intelligence gave sustained pressure towards powerful modern language, with large vocabularies and complex syntax. Sexual selection is a self-reinforcing process, which may have been kick-started by some habitat-related need such as hunting.

Language then evolved as the fastest and most efficient way to display intelligence. Every conversation since the dawn of language has had the hidden covert goal of displaying intelligence, as well as its overt practical goals. This has led through gradual stages over 2 million years to the modern highly capable language ability; for most of that time, language has been faster and more capable than is needed for survival purposes. Its power and speed have been used for the covert purpose of displaying intelligence—to compete for mates.

It is not possible from the fossil record to trace the course of evolution of our language ability, from some early proto-language to its present capability. The account from sexual selection is consistent with many different accounts, such as that in Bickerton (2014) or Botha (2016). It is a virtue of the sexual selection account that it does not conflict with those accounts, but merely adds an extra, strong selection pressure to drive the process—particularly in its later stages. However, the need to display intelligence does motivate one possible account of the course of language development, described in section “Stages in the evolution of language,” in which pragmatic skills—the ability to exchange knowledge over extended dialogues—came before any syntactic abilities.

It is a characteristic of sexual selection that, once a species has embarked on a trajectory of sexual selection for some trait, all the further steps along that trajectory are unique to that species—because the selection pressures for each stage do not act on members of any other species. In this case, for reasons that we cannot know for certain (but which may concern the need for an early form of language, for instance to organise hunts or make tools) early *homo* embarked on a trajectory of sexual selection for superior intelligence, with language as the main display mechanism. Once this process had started, all the subsequent stages, as described in this paper—including the refinement of pragmatics, the development of spoken symbols, and the development of syntax—were inevitably restricted to *homo*, and not shared by other great apes; and within *homo*, they were driven by the need to display superior intelligence.

The same principle applies to any physical or neural adaptations required for high intelligence, or for the fast language needed to display it—such as an enlarged brain, changes to the larynx, and other changes (Lieberman, 2006). These happened in *homo*, and in *homo* alone, because there was no selection pressure for them to happen in any other species. Of the physical changes, possibly the most expensive in survival terms were the extra metabolic costs of a larger brain—which for any individual, were offset by the reproductive benefits.

## The theory of sexual selection

I distinguish between natural selection—the need to adapt to environmental challenges in order to survive to adulthood—and sexual selection, which is the need to find a mate, to pass on your genes.

Sexual selection is a very common phenomenon. It is responsible for much of the visible diversity of life (such as flowering plants, and tropical birds’ plumage) for much animal behaviour, and is arguably the main driving force for the great diversity of species. It is hard to find any animal species which has not been shaped by sexual selection.

Yet in a recent collection of 17 articles by leading authors on the evolution of language (Christiansen and Kirby, 2005), the phrase “sexual selection” hardly appears. Every chapter in the volume takes it as a given that language is a beneficial adaptation, arising from natural selection. However, many of the traits of human language, which, as above, pose difficulties for any account by natural selection, fit easily with an account of language by sexual selection.

Whenever a species has a trait which is unique to that species (not seen in related species in the same habitat), and which is exaggerated beyond any natural need, it probably indicates that the trait evolved by sexual selection.

The paradigm case of sexual selection (Lande, 1981; Maynard-Smith, 1982) is the peacock’s tail. Male peacocks have large tails, which may reduce their individual fitness. Female peahens find the large tails attractive. While this may diminishes the overall survival fitness of the peacock species, it is effectively locked in by a conspiracy between separate genes expressed in the two sexes. These are the genes which determine some property of the phenotype (in one sex) and genes which determine the preference for that property (in the other sex):

- If a male peacock had a shorter tail, he would survive better, but would be unable to find a mate; so his genes would die out.
- If a female peahen found shorter tails attractive, her male offspring would be less able to find mates; so her genes would die out.

This has been analysed mathematically, using the tools of evolutionary game theory and population genetics (Lande, 1981; Maynard-Smith, 1982). Without the female influence, the male-expressed genes would evolve towards an optimum tail size for survival. However, the female-expressed genes do not evolve towards preferring the best tail size; they prefer a bigger tail size. Then any male with a tail size bigger than his peers will succeed in the reproductive competition.

This results in an evolutionary arms race amongst males, with strong selection pressure towards larger tail sizes, which may be less fit (although this point is controversial—Askew, 2014). The race only stops at the tail size where the incremental loss of survival from having a larger tail balances the incremental gains in reproductive success. This point of balance is called the Evolutionary Stable Strategy (ESS; Maynard-Smith, 1982). A species can stay stably at that point over many generations.

Sexual selection is a process of runaway positive feedback, between male-expressed genes and female-expressed genes, which amplifies small departures from the point of greatest individual fitness, and then locks them in to the species at a stable point, the ESS, which is not the optimum point of individual fitness; so it may involve handicaps. It can involve sexually selected traits expressed in both genders. There are several complex models of mate choice (Kokko et al., 2003, 2006); but typically, sexually selected traits must be visible to potential mates, to influence mate choice.

Since the mathematical analysis of sexual selection in the 1980s, the role of handicaps in sexual selection has been controversial. It is now recognised (Számadó, 2011; Penn and Számadó, 2020) that there is no general “handicap principle” as proposed by Zahavi (1975); or if there is any such principle, it has been widely misunderstood, and its theoretical basis has been misunderstood. In my discussion of sexual selection for human language and intelligence, I shall not rely on any universal handicap principle or its theoretical underpinnings.

I shall instead rely only on some empirical facts about mankind: that superior intelligence requires both a larger brain with greater energy costs, and a longer period of parental care; and that these can reduce survival, by requiring more food, and diverting parents from other survival-related activities, and making infants vulnerable for longer times.

**Figure 2:**
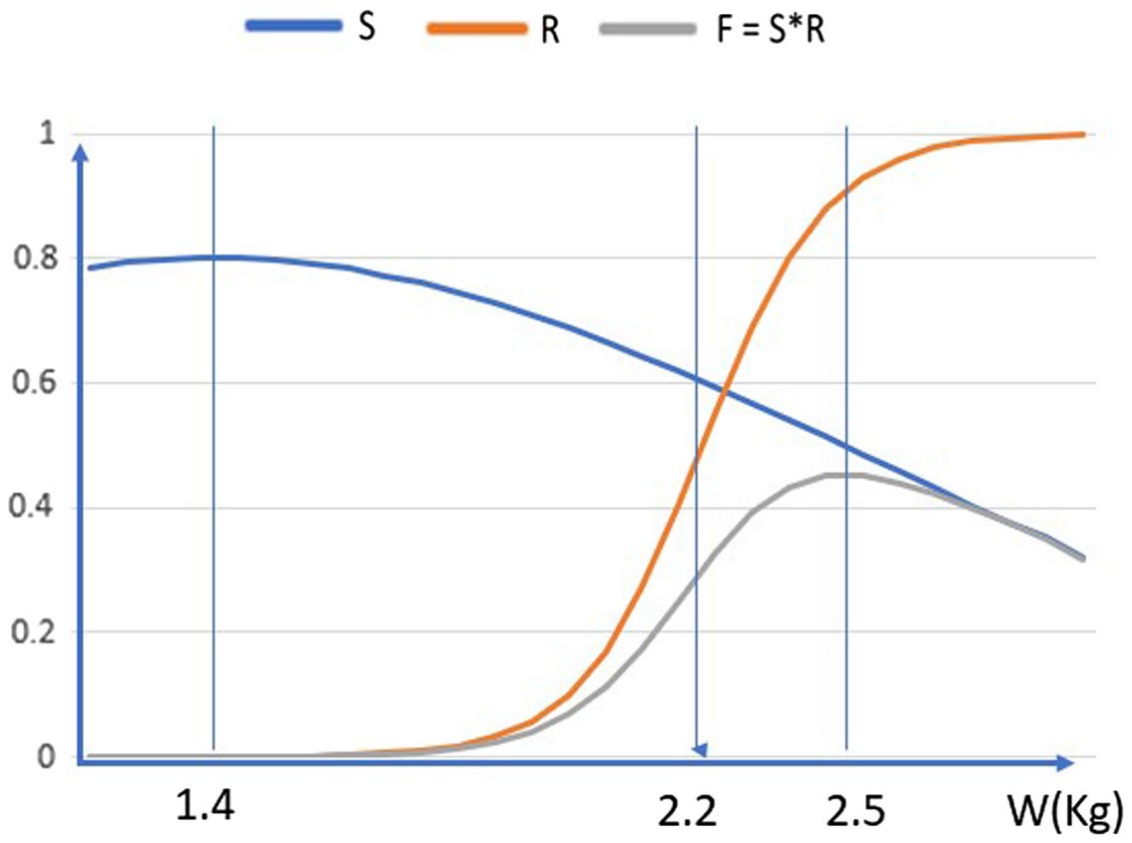
Illustration of sexual selection for size of the human brain. The optimum point for an individual is where the probability of surviving to adulthood and finding a mate (S*R) is maximized. The optimum point moves to the right over evolutionary time, increasing the handicap in survival fitness from sexual selection.

In the case of human language, this paper proposes that at some stages of its evolution, language evolved as a display of superior intelligence, and that the main survival handicaps of greater intelligence are empirically as described above. How this leads to reduced overall fitness can be illustrated graphically (see Figure 2), as follows:

- Fitness F is defined as survival S times reproduction R.
- The vertical axis shows probabilities F, S, and R in the range 0.1. These are shown as a function of brain weight W, in kilogrammes—which is the horizontal axis,
- The probability of an individual surviving to adulthood is denoted by S, and is modelled as Gaussian function exp.[−(W–W_0_)2/σ^2^], with maximum probability 0.8 at a weight W_0_ = 1.4 Kg, and decreasing at higher brain weights, because of the empirical factors described above—not because of any general handicap principle.
- The probability of an adult individual finding a mate and reproducing is denoted by R, and is modelled as a sigmoid function 1/(1 + e^-λx^). This “competitive” function is small when the brain weight is small (so relatively unintelligent people do not find mates), rises up to 0.5 at W = 2.2Kg, and then rises further towards 1.0 for higher brain weights.
- Each individual needs to pass on its genes as many times as possible, by maximising F = S*R. This is the probability of surviving to adulthood and then reproducing.

The optimum point for an individual—the brain weight that is selected, by combined natural and sexual selection, to maximise F = S*R—is about 2.5 Kg. Both lower brain weights and higher brain weights give smaller S*R than this value.

At this weight, the survival S is reduced to about half of the maximum it has at the “habitat-ideal” brain weight of 1.4 Kg. These figures are merely illustrative of the points that (a) in the case of human intelligence, sexual selection reduces individual fitness; and (b) it leads to traits, such as intelligence and brain weight, that are exaggerated beyond any survival-related need. The precise forms of the curves are presumably different from those shown; but still we would expect the product S*R to have a maximum at some weight larger than the weight which maximises S.

Sexual selection involves large selection pressures of reproductive competition, and so happens rapidly when it happens. Unlike the gradual evolution of traits which determine survival—which is a slow convergence towards a moving optimum—sexual selection is a more all-or-nothing affair. You may have the best genes for survival in the habitat, but if you do not find a mate, your genes are out of the game. That, and the runaway positive feedback between two sets of genes (for a visible trait, and for the preference for that trait) leads us to expect that changes to a species by sexual selection will be (in evolutionary terms) a comparatively rapid process.

Because sexual selection is a positive feedback process, its results are arbitrary and species-specific. When there is sexual selection in a set of traits—which is like a movement in the several dimensions of the traits—then the point of greatest survival fitness in those traits is like the peak of a hill, in a multi-dimensional space. If the species is initially like a ball balanced on that peak, then as soon as the ball starts to roll in any direction, the runaway positive feedback of sexual selection reinforces the movement, in whatever direction it started. That direction is arbitrary, and there is no way to predict that it will start to move in one direction rather than any other; but once it starts, it is a runaway positive feedback effect.

For instance, many species of bird have elaborate decorative plumage. This has costs in individual survival fitness (dull, camouflaged plumage would be fitter) and is a sexually selected trait. So different species have different plumage, but the details of each species’ plumage cannot be predicted or retrospectively explained. The results of sexual selection have an arbitrary, unpredictable character.

In summary, this analysis shows how sexual selection for greater intelligence can lead to traits which:

- Evolve relatively rapidly, by positive feedback between the sexes
- Are arbitrary and species-specific
- Are exaggerated beyond any habitat-related need
- May reduce the survival of an individual.

To be sexually selected, superior intelligence must be signalled to potential mates. This paper proposes that language evolved in part as the most efficient signal of superior intelligence.

## Intelligence and language evolved by sexual selection

Humans differ from other primate species not only in language, but also in our larger brains and greater general intelligence. This suggests that the combination of superior intelligence and language may have evolved together. This paper proposes specifically that language is the display through which superior intelligence has evolved.

Miller (2001) has given evidence that human intelligence evolved by sexual selection. Our large brains show the hallmarks of sexual selection—such as rapid evolution, species-specificity, and exaggeration, to the point of reducing individual survival—in the huge energy requirements of the human brain, increased size of the birth canal, and extended period of immaturity. The fossil record shows that our brains have been large enough reduce individual survival for more than 2 million years.

Sexual selection cannot take place without display of the sexually selected traits (so they can affect mate selection); in order to drive selection, the traits must be visible to potential mates. So if there is sexual selection for greater intelligence, we must have ways to display our intelligence to potential partners.

If we could only display greater intelligence by making a better spear or shelter, or by cooking a tastier rabbit, that would be a slow and inefficient form of display (and it could sometimes be copied or faked). Sexual selection for intelligence will only work if there is some rapid and reliable, hard-to-fake display of intelligence. Language is that display.

Miller (2001) identified that the evolution of language involved sexual selection. In this regard Miller regards language as one of a number of ways of displaying intelligence—alongside other cultural displays such as art, invention, ritual and dance. He does not, as this paper does, treat language as the pre-eminent display of intelligence. It is so, mainly because of its much greater ability to convey information than other channels.

Language is something we can rapidly display at any time of the day, addressing any topic; and novel appropriate language is hard to copy. As such, language can be a very effective and authentic display of intelligence. This paper proposes that sexual selection for intelligence depends on sexual selection for language. However, this may have come about in several stages, some of which came before spoken language.

In order to display intelligence to someone, you need to convey information to them, which they recognise could only have been created by the use of intelligence. How can they know that the message you convey to them is an individual message, and not somehow copied, or pre-fabricated? They can only know this (and know that the information is correct, and is not just made-up noise) if the information relates to something that they already know—so they can check it.

Hence, to send a verifiably intelligent signal to someone, you must first know something about what they know—to relate your message to something they know and can check. To display intelligence, you need the beginnings of a theory of mind (ToM).

So the first requirements for the display of greater intelligence are:

- A theory of mind, to represent internally what the other person knows, so you can build on it
- Iterated exchanges of information, to convey information in both directions
- Acknowledgements, and the recognition of acknowledgements, to know what the other person has understood at any stage of the exchange.

The starting point for knowing what somebody else knows is to recognise a “common ground” between you and them (Clark and Wilkes-Gibbs, 1986; Clark, 1996; Stalnaker, 1999; Stalnaker, 2002; Tomasello, 2003). The common ground consists of what you both know, either because you can both see the same things, or can make the same inferences from what you see, or because you share some recent experiences, or because you have some shared skills or goals or assumptions. It grows as you exchange information.

We can now start to discern the rules of the game by which you can display superior intelligence to another person. You first recognise the common ground of information you share with them. You then add information to that common ground (referring to it), which they can understand and sometimes check. They in turn add information to the common ground; and as you receive that information you can check if they have understood your message (assessing their intelligence, as well as assessing your own success in sending them information). This proceeds in a series of iterative information exchanges, adding information to the common ground. If the information in the common ground continues to grow, you both know that the interaction is succeeding.

For every type of sexual selection, there is some measure of success which the participants are trying to achieve—some score they are trying to maximise, which is defined innately in their brains, and which determines their reproductive success. For peacocks, this score is mainly the perceived size of a tail. For some birds, it is the match of their plumage to a genetically determined attractive pattern. For animals which have a mating ritual or dance, the score is the conformance of the dance to some arbitrary genetically defined pattern. What is the score to be maximised for the human display of intelligence?

It is not easy to design a reliable measure of superior intelligence, which cannot be faked. The design space for such a measure is limited. As described above, it depends on information you impart to another person, and it must be possible for them to check the information you impart, so that information must relate to their own knowledge. This requires both a theory of mind to represent their knowledge, and extended cooperative sequences of information exchanges, to find out what they know and relate your contributions to it.

I suggest that the score for display of intelligence is some quasi-multiplicative combination of:

- The frequency of occasions on which information-sharing exchanges can take place
- The amount of verifiable new information added to the common ground, by each step in an exchange
- The relevance of the new information to what the participants already know
- The diversity of the inferences used to infer the relevance of new information
- The speed at which exchanges take place.

This scoring system will have been built into our minds genetically over the whole period of sexual selection for intelligence. It did not happen all at once; different factors could have come into the scoring formula at different evolutionary eras.

Note that, as we can convey information by our actions, any of the five scoring factors above could have evolved before spoken language.

The score accumulates not only through encounters with potential mates. Scoring highly with any cooperating partner can be a mark of high social status, or high rank in the group—which in primates, leads to reproductive success (e.g. Worden, 1996). So the selection pressure can more accurately be termed “sexual and social selection;” the two are completely entwined. Achieving high social status is a lifelong endeavour—not just a mating activity.

In summary, you can display intelligence—and succeed in the sexual/social selection game—by achieving high scores in an iterative information exchange game, with scores defined as above. The key skill required to get high scores is the skill of dialogue pragmatics—carrying out extended cooperative information exchanges.

In this picture, pragmatics is not just a poor cousin of the better studied “central” topics of language like syntax and semantics. Pragmatics and the Theory of Mind are where language begins.

## Stages in the evolution of language

As in the previous section, the covert goal of any person in an information exchange is to show off their superior intelligence (as well as achieve any overt practical goal of the exchange). The primary skill that this requires is the skill of understanding what your conversational partner knows, over a series of turns in an exchange, to relate your contributions to what they know. This skill relates to language pragmatics, rather than to syntax or semantics. It leads to a possible model of the evolution of language in which pragmatic skills evolved before syntax and semantics.

This section describes such a model. It is proposed as just one possible model; there are of course difficulties in validating any model from the evidence available.

In the examples which follow, I have not explicitly emphasised that each example is an example of sexual selection. However, they are all examples of sexual selection in action. The reader should be aware that for each example, as well as the overt purpose of each participant, both participants have the covert goal of displaying their intelligence to the other; and that a successful cooperative exchange achieves this goal. For instance, just by carrying out any exchange, each participant demonstrates some Theory of Mind abilities.

### Pragmatics before language

If we define pragmatics as the study of cooperative information exchanges, adding information to a shared common ground, and scoring success by the five criteria listed above, then pragmatics are possible before any language exists. Consider the following wordless exchange:

A: <gets saucepan out>
B: <gets eggs out>
A: <smiles, takes eggs>

Here, B recognised that eggs were relevant to A’s action of taking the saucepan out. This was because they both know a procedural cooking script, or frame (Schank and Abelson, 1977; Fillmore, 1985) with steps:

- <get saucepan>
- <get eggs>
- <boil eggs>
- <eat eggs>

Such a script can be represented as a tree-like feature structure, of the kind that have been used in the computational modelling of language for many years (Kaplan and Bresnan, 1981; Gazdar et al., 1985; Shieber, 1986).

So when B saw A’s action of getting the saucepan out, B recognised A’s intention to eat eggs—a Theory of Mind inference. This made B’s action relevant to A. In more detail, the inferences made by B are:

- See A’s act: <get saucepan out>
- Search for procedural scripts that include getting out a saucepan
- Find the “boil eggs” script
- Does A know that script? Yes (ToM step)
- Is it the best-fit script (using a saucepan) that A knows? Yes.
- So A intends to eat eggs
- So the “boil eggs” script is relevant to A
- So other objects in that script are relevant to A
- So eggs are relevant to A

Similarly, A already has the “boil eggs” script in his mind (because that is his current plan)—so A can immediately see the relevance of B’s action. Recognising B’s intention to help him, A acknowledges by smiling, and takes the eggs from B. A and B both know that the interaction has been successful, and they have both scored points in the “display your intelligence” game.

Note that this simple exchange shows all the features of information exchanges identified in the previous section—iterative exchange of information, acknowledgement of information, and a theory of mind.

Participants can play this interaction game, and score sexual selection points, before they have any language. Actions convey information, and that information is relevant (and so scores points) if it can be related by some inference to information in the common ground. Actions can include pointing, directing gaze, mime, and showing—which are part of infants’ earliest social interactions and are the foundation of deixis.

So Sperber and Wilson’s (1986) Relevance-based theory of pragmatics (Sperber and Wilson, 1986; Carston, 2002; Wilson and Sperber, 2012) could have had its origins before language, in sexual and social selection for intelligence. A possible pre-linguistic origin for relevance-based pragmatics has been noted by Sperber (1994) and Origgi and Sperber (2000).

If the procedure for boiling an egg is represented in the minds of both participants (and so is part of their common ground) as a feature structure, and the individual acts like “pick up saucepan” are represented as feature structures, then the key operation needed to infer the relevance of some act to something in the common ground is the operation of unification. Therefore two of the key features of many computational models of language (feature structures and unification) are already useful for pre-linguistic pragmatics. Feature structures and unification are both necessary for primate social intelligence (Worden, 1996).

Grice’s (1989) principle of cooperation can be motivated by need to cooperate to score points in the interaction game, even before language exists. The innate scoring system becomes the scoring system for all of Wittgenstein’s (1953) language games.

### The emergence of symbols

If people can learn and convey the meanings of symbols (Deacon, 1997), such as single words (or any unproductive constructions, in the sense of Cognitive Linguistics; Langacker, 1987; Langacker, 1991; Goldberg, 1995; Feldman, 2008), they can make the interactions faster and more frequently usable, and so can score more points. A spoken symbol can be faster than doing a real act or picking out a thing, if the thing is not to hand. As the interaction becomes faster, it requires more intelligence.

For instance, if A’s first action is ambiguous, because several things can be boiled:

A: <gets saucepan out>
B: eggs?
A: no
B: cabbage?
A: yes
B: <gets cabbage out>
A: <smiles, takes cabbage>

This interaction can work in a wider range of situations than the first interaction will work in – even when saucepans have many uses – and is faster than B laboriously getting out all the cookable things. The better B can guess what A intends to eat, the more B can demonstrate intelligence (by making the exchange faster). The introduction of a vocabulary of spoken symbols (or other constructions) both requires more intelligence, and creates more opportunities to display it. This is a further step in the sexual selection arms race.

By taking their turns in these exchanges, both participants add information to the common ground they share. So non-verbal pragmatics evolves to single-word, or single construction, semantics.

As in the pre-linguistic stage, one quick way to recognise the relevance of some word to the common ground is to unify the meaning of the word with some feature structure in the common ground (in this case, unify with the script for the procedure of boiling cabbage). Pragmatics at the one-word stage uses unification of word feature structures with feature structures in the common ground, to infer the relevance of words. Words refer to the common ground, which enriches their meaning by unification (for instance “he” may refer to some individual in the common ground, and acquires the meaning of that individual by unification).

In this way we can trace the beginnings of language—as far as a one-word stage, which all infants pass through—through the sexual selection imperative to display intelligence by scoring highly in the pragmatic information exchange game.

It may be worth repeating that the transition from pragmatics to symbols took place in *homo*, and not in other great apes, because when this transition took place, only *homo* had embarked on a trajectory of sexual selection for the display of superior intelligence—so other great apes did not have the pragmatic basis, or any selection pressure, to develop symbols in the same way.

### Syntax emerged from pragmatics

Consider an evolutionary era when humans have evolved the ability to carry out extended dialogues, using repertoires of learned discrete (unproductive) symbols, with learned pragmatic rules to define, at any stage in a dialogue: “how symbol X is relevant to the common ground.” By engaging in open-ended iterative dialogues of this kind, one person can convey a discrete infinity of meanings to another person. The “language rubicon” of a discrete infinity of meanings (Hauser et al., 2002) can be crossed by pragmatic inference rules, with no grammar.

Then, grammar can emerge as a codification of pragmatic relevance rules, to make the exchanges faster, and so to score more points for sexual selection.

Suppose that the participants have learnt the meanings of some unproductive words for actions, and some words for things. This enables a simple request:

A: pass
B: what?
A: eggs
B: <passes eggs>

Here the word “what?” means: “You have just described an act you want me to do. What thing do you want me to do the act with?” So “what” will be very commonly used—it is so common, that it can often be omitted, shortening the dialogue:

A: pass
A: eggs
B: <passes eggs>

Now A is saying “pass eggs,” and the verb “pass” starts to become productive—being able to combine with any thing-denoting word to convey a wide range of meanings in fewer turns of an exchange—that is, faster, and scoring more points.

Initially, B understands what A says by first adding the meaning of “pass” to the common ground, then unifying the meaning of “eggs” with the feature structure for “pass” in the common ground. Later, B may directly unify feature structures for “pass” and “eggs” within the utterance “pass eggs,” before adding anything to the common ground. This is the parsing process of a unification-based grammar (Kaplan and Bresnan, 1981; Gazdar et al., 1985; Shieber, 1986), which emerges as a streamlining of pragmatic inferences.

This simple “pass eggs” example can be the start of VO word order; initially in a “verb island” manner (Tomasello, 2003) for specific action words, and later moving to more general grammatical patterns; or to larger patterns, such as the ditransitive “pass eggs Mum.” This could have been the evolutionary origin of syntax (Christiansen and Chater, 2016).

It is not yet clear how far this transition from pragmatics to syntax can be taken; but it seems that there is no obvious limit to the number of pragmatic sequences that can be learned, of the form: “If I say X, and then I say Y, then this is how Y is relevant to X”; and can then be transferred into a syntactic rule for the meaning of XY. Pragmatic inference rules crystallise to grammatical rules. This possible origin of syntax from relevance-based pragmatics has been described by Origgi and Sperber (2000).

In this way, the tidier parts of pragmatics can condense out to syntactic rules—leaving only ugly individual facts to remain part of pragmatics. There is no limit to these ugly individual facts (Ariel, 2017)—and there is no need to limit their number, because people can learn very large numbers of them. Knowing many pragmatic rules is a sign of superior intelligence, so the rules are expected to proliferate. This means that there is no clear boundary between pragmatics and syntax—because the neater parts of pragmatics are on their way to becoming syntax.

In this way, the selection pressure to exchange information as fast as possible leads to the emergence of language in the order:

Pragmatics = > word meanings = > grammar.

We will never learn from the fossil record the true order in which language evolved; but this order for the evolutionary emergence of language can be motivated by two reasons:

- This is the order in which infants acquire language: there is strong evidence that babies have some pragmatic skills before they know any words; that they need these skills, including a theory of mind ability, to learn word meanings (Bloom, 2000); and that only after that, they start to learn syntax (Tomasello, 2003). On an assumption that ontogeny often retraces phylogeny, the capacity for language may have evolved in the same order.
- This order of emergence of language builds outwards from existing primate cognitive abilities, requiring no large discontinuities—not requiring too much new design information in the human brain at any stage (Worden, 1995, 1998, 2022a). We know that chimps have some limited theory of mind, including a limited appreciation of what is and is not in the common ground (Tomasello and Call, 1997; Call and Tomasello, 2008; e.g., knowing what things another chimp cannot see). Then, learning individual word meanings requires only a Bayesian learning ability close to that used for ordinary associative conditioning in many animals (Anderson, 1990); finally, early syntax can be learnt using a modest extension of Bayesian learning (Worden, 2022b), to learn and use at least a simple unification-based grammar.

The progression through these three stages is best motivated by sexual/social selection—the need to exchange information as fast as possible, to score points in the competition for social status. This depends on the strong selection pressure of reproductive success—if you do not win the competition, your genes are not passed on. Without that strong selection pressure, the gains in fitness arising from very fast information exchange would be much smaller, and are not sufficient to offset the costs of a larger brain.

While this model of language has a role for a human theory of mind in language—in inferring what another person knows (Sperber and Wilson, 2002), so that your communication can display your intelligence to them—it may remove the need for the many nested levels of meta-representation which have been proposed by Grice and others—as in deeply nested statements like “*the communicator must intend her audience to believe that she intends them to believe a certain set of propositions*” (Sperber, 1994; Stalnaker, 2002). This four-level nesting of states of mind is computationally complex, and seems to be unnecessary most of the time; in this model of language, it is usually not necessary.

When you start a conversation with someone, you engage in an interaction game, whose scoring system requires you to behave as if the interaction could be described in an elaborate nested theory-of-mind manner. That is how your brain innately measures your score, and requires you to play the game. But some of the more deeply nested ToM computations need not take place; or they can happen only selectively, when default assumptions are violated. The scoring system which guides this is a part of your innate mental heritage, but it is not in your conscious awareness.

### Neurobiological foundations for language

Many authors have studied the neurobiological foundations for language, and the question arises: how, in this hybrid account of the evolution of language, did those foundations evolve? This question is distinct from the question of how more abstract language capabilities such as pragmatics and syntax evolved (Mondal, 2019). Many authors place emphasis on spoken language, and the physical and neurobiological adaptations required for it. This model of language evolution suggests a rather different emphasis.

The first requirement for the display of superior intelligence to your peers is an enhancement of existing primate Theory of Mind capabilities, to appreciate what others know, and how they will regard your communications. The first stage in enhancing the human theory of mind may have been the need to understand a “common ground” of mutually known facts, for cooperative activities such as hunting. As described in section “Pragmatics before language,” this led on to the skills of pragmatics. So the first requirement for language was an enhanced Theory of Mind capability. There has been little research on where this is located in the brain; but since it requires integration of many modalities of information, it may possibly be associated with enlargement of the pre-frontal cortex. Patients with frontal lobe or temporoparietal junction lesions find some Theory of Mind tasks difficult.

Moving beyond pragmatics, we come to the use of large numbers of symbols (spoken or otherwise) as in section “The emergence of symbols,” and syntax, as in section “Syntax emerged from pragmatics.” Both of these were arguably the “supercharging” of existing primate capabilities; for the first, to learn up to hundreds of symbols (as in when primates have been trained in languages); and for the second, many computational models of syntax rely on unification, which is arguably a pre-existing requirement for primate social intelligence (Worden, 1996). Symbols and syntax may well have required more modest and specific extensions to existing primate capabilities, than the wide-ranging and generic Theory of Mind required.

Finally, we may consider the sensory-motor capabilities needs for language; fast articulation through a modified larynx, and other language-related adaptations. In one sense, these are not exceptional. Many animal species develop outstanding sensory-motor capabilities for their niches (for instance, the mongoose to read snakes’ movements and to strike; or swallows or bats, to catch small insects at speed). These all require highly specialised neural and physical adaptations. The topic of human sensory-motor adaptations for language, while being of considerable interest, is not the primary focus of this paper.

## Three phases in the evolution of language

It may be useful to identify three phases in the development of modern language:

- The first phase, from the early enlargement of the human brain about 2 million years ago, when early language arose as a response to habitat needs such as cooperative hunting
- The second phase, when sexual selection for superior intelligence began, and its runaway selection process led to powerful language, high intelligence and enlarged brains
- The third phase, from 10,000 years ago to the present day.

We cannot find any trace of language in the fossil record, and we cannot put precise dates on the evolutionary emergence of language in the first two phases. However, on the hybrid sexual selection theory, any rapid enlargement of the human brain was probably driven by sexual selection; so the second phase may possibly have started about 1 million years ago.

Miller (2001) proposed that language and other cultural displays evolved by sexual selection for superior intelligence. We know that:

- *Homo* has had an enlarged brain, with some handicap of its extra metabolic costs, for more than 2 million years (DeSilva et al., 2021); and this handicap accelerated about 1 million years ago.
- From 2 million years ago until 10,000 years ago, superior intelligence did not give mankind much extra fitness, as measured by human population size (Harpending et al., 1998).

These facts are consistent with superior intelligence as a sexually selected trait. They imply that sexual selection for intelligence may have been happening for something like 1 million years.

Miller (2001) has identified many ways in which superior human intelligence can be displayed, including complex behaviour, ritual, art, dance, music, invention, and conversation. Before about 200,000 years ago, there is no evidence for any of them in the fossil record (except for stone tool-making, fire and cooking). This leaves open the question: which display of superior intelligence drove its evolution from about 1 million years ago to 200,000 years ago? There must have been some display of intelligence; without it, sexual selection for intelligence could not have happened.

In an open field for displays of intelligence, language stands out as the most convenient (we can do it at any moment in the day), and as having the highest bandwidth—we can display intelligence faster through language than through any other medium—even before language came to its present supercharged form. A case can be made that over a period of 1 million years, language has been the primary display and driver of sexual selection for intelligence. This was the second phase in the evolution of language.

In the first phase, early language may have been driven primarily by natural selection, for instance from its benefits in hunting or tool-making. During the second phase, the increasing power and speed of language did little to increase our survival fitness—not enough to compensate for the penalties of having a larger brain; and in terms of the practical, life-enhancing uses of language, its increasing power was largely wasted. This was because there were not many interesting things to talk about – mainly, daily social gossip (Dunbar, 1993) or occasional social commitments (Deacon, 1997). Meanwhile, language served its covert, competitive purpose of regulating social and reproductive competition—driving mankind in a runaway selection process towards greater, and biologically superfluous, intelligence. People used language not only to persuade other people of their ideas (Mercier and Sperber, 2017) but also to impress other people by their intelligence.

It was only about 10,000 years ago, with the emergence of agriculture and settled communities, that human intelligence and language found its proper role, in the third phase of evolution of language. Settled communities supported specialisation and barter, increasing the complexity and interest of human lives. Finally, powerful language found its modern role—enabling us to think and talk about the interesting things and institutions we have created, and so enhancing our lives.

During this third phase of language evolution, societies have changed rapidly, and languages have changed to meet their changing needs. This has been a different form of language evolution (Worden, 2002; Dediu et al., 2013), by the evolution of language constructions, rather than the evolution of people; over the same period, human intelligence and our capability for language has hardly changed at all.

## Evolution of the brain

Animals with complex brains have existed for more than 500 million years, since complex sense organs appeared before the Cambrian Explosion; but the enlarged human brain and our capacity for language have existed for less than 2 million years. This suggests that while our brains have recently greatly enlarged, the amount of new design information in the brain—including any new brain design required for language—may have been much more limited, perhaps being of the order of 2/500 = 0.4% of all brain design.

This view is supported by a speed limit for evolution (Worden, 1995, 2022a,b) which limits the rate at which new information expressed in any part of the phenotype (called “Genetic Information in the Phenotype, or GIP”) can increase by evolution. The speed limit implies that the rate of increase in GIP of the human brain has been less than one bit per generation—which would only allow a few kilobytes of new brain design since the dawn of language.

Mankind could have acquired the capability for language without major design innovations in the brain, in the following manner: in cognitive linguistics (Langacker, 1987, 1991; Goldberg, 1995), language is the use of constructions, which can be represented as composite feature structures, and are used in language by the operation of unification (Kay, 2002). Unification-based computational models of language have been used for many years (Kaplan and Bresnan, 1981; Gazdar et al., 1985; Shieber, 1986). They can be used to build working computational models of simple language use and learning (Worden, 2022b). However, both feature structures and unification may pre-date mankind—being used for many millions of years to support primate intelligence (Tomasello and Call, 1997), particularly primate social intelligence (Worden, 1996).

Hence, if language depends on feature structures and unification, it may not have required any major innovations in brain design—mainly just the enlargement of some existing parts of the brain to make them faster and more powerful (e.g., to support large vocabularies), which is consistent with the speed limit for evolution.

This cognitive linguistic picture of the evolution of language in the brain contrasts with the model from generative linguistics, particularly in its recent minimalist versions. Specifically, the Chomskyan “Prometheus” model, of language evolution in a single evolutionary event, would violate the speed limit for evolution.

Chomsky (2002, 2010) maintains that the “language organ” resulted from a single genetic mutation, probably within the last 100,000 years: “*Within some small group from which we are descended, a rewiring of the brain took place in some individual, call him Prometheus, yielding the operation of unbounded Merge, applying to concepts with intricate (and little understood) properties… Prometheus’s language provides him with an intricate array of structured expressions with interpretations of the kind illustrated: duality of semantics, operator-variable constructions*…”

In this account, elaborated by Bolhuis et al. (2014), the key innovation in brain design required for productive human language was the merge operation, which enables recursive grammar, and is required to express a discrete infinity of possible meanings in one utterance. On the account of Bolhuis et al., the evolution of the merge operation occurred within a very short timescale: “*the language faculty is an extremely recent acquisition in our lineage, and it was acquired not in the context of slow, gradual modification of preexisting systems under natural selection but in a single, rapid, emergent event that built upon those prior systems but was not predicted by them.*.”

While Bolhuis et al. say that the change required to introduce the merge operation was “relatively minor,” it was nevertheless a “single, rapid, emergent event” occurring less than 200,000 years ago. Since the breeding population of homo sapiens at this time was of order 10,000 (Harpending et al., 1998), or 2^13^ people, the chances of a single evolutionary event in a single individual producing as much as, say, 24 bits of appropriate new design information in the brain are of the order of 2^13^ * 2^−24^ = 2^−11^, which is a chance of one in a hundred billion.

If an evolutionary account requires any event with probability less than one in a billion, it can safely be dismissed. On the other hand, if the innovation for the merge operation required less than 24 bits of GIP, that would be remarkably little new brain design for the crucial operation of merge, which has been so important for the human species. So all forms of the “single evolutionary event” account can be rejected.

## Comparison of scenarios for the evolution of language

Számadó and Szathmáry (2006) have reviewed a range of scenarios for the evolution of language, and have proposed criteria for evaluating the different scenarios. They list a number of possible accounts (primary uses of early language) which have been proposed for language evolution:

- Gossip
- Grooming
- Group bonding/ritual
- Hunting
- Language as a mental tool
- Mating contract and/or pair bonding
- Motherese
- Sexual selection
- Song hypothesis
- Status for information
- Tool making

They identify four questions to ask of each hypothesis:

Honesty: can the theory account for the honesty of early language? (note: this question relates to the honest use of language, to say true things – not to any concept of “honest signalling” as in sexual selection).
Groundedness: are the concepts proposed by the theory grounded in reality?
Power of Generalisation: can the theory account for the power of generalisation, which is unique to human language?
Uniqueness: can the theory account for the uniqueness of human language?

These questions provide principled criteria to assess the relative strengths of different theories, and this comparative approach is an important contribution to the field. In their table 1, Számadó and Szathmáry find that only two of the hypotheses (hunting and tool making) can answer “yes” to the first three questions; and that none of the 11 hypotheses can positively answer the fourth “uniqueness” question. Their answers apply not only to the papers they cite, dated before 2006; they apply to later hypotheses such as preparation for hunting (Számadó, 2010) or competitive scavenging (Bickerton and Szathmáry, 2011).

For the sexual selection hypothesis, Számadó and Szathmáry answer “no” to all four questions. In support of these answers, they cite Miller (2001). However, the main focus of Miller’s book “The Mating Mind” was not language; language was only one of several displays of intelligence, and he devoted little detailed discussion to how language evolved. The book gave little information to answer the questions positively. From the more detailed considerations of this paper, we can re-visit the answers to the four questions for the sexual selection hypothesis:

Groundedness—Yes: In order to display superior intelligence, you can exchange information about any topic—including concrete topics which can be grounded by pointing at real objects, and by other ostensive actions. For purposes of sexual selection for the display of intelligence, it is not necessary to talk only about abstract sex-related or person-related topics; these are optional, as additions to concrete language.
Honesty—Yes: If every conversation has the covert goal of reliably displaying superior intelligence (driven by sexual selection), then it requires an extended sequence of exchanges, to know what the other person knows, so they can verify that your own contributions are the result of intelligence. If your contributions are factually wrong, they will not be taken as a sign of intelligence—quite the reverse. If an exchange breaks down through false information, neither participant gains through the display of intelligence. This provides a sufficient incentive for the Gricean cooperation and level of honesty that we observe in human language today—and which we assume, in the absence of direct evidence, occurred in earlier language use. Sexual selection for intelligence is not restricted to talking about topics where conflicts of interest arise—such as sexual fidelity.
Power of Generalisation—Yes: If the driving force for evolution of the language capacity is the need to display superior intelligence, the ability to generalise will surely be seen as a sign of intelligence, by those peers who have the intelligence to appreciate it. Even when they do not, the power to generalise enables you to say new and true (and therefore impressive) things about any topic.
Uniqueness—Yes: While sexual selection can lead to attributes which are common across species, such as male–female size disparities, there are many examples (such as the plumage of tropical birds and the colouring of flowers) where the results of sexual selection are unique to one species, and are not related to survival requirements. On this account, humanity happened to embark on a trajectory of the display of intelligence to attract mates, while other ape species did not. Sexual selection enhances traits by runaway positive feedback. Language is the resulting species-unique way to display intelligence.

So the sexual selection hypothesis now gives positive answers to all four questions. It is the only account which answers the uniqueness question.

As well as Számadó and Szathmáry’s (2006) four questions, I suggest a fifth question to ask of any account of language evolution:

Selection Pressure: How strong is the selection pressure implied by the primary purpose of language in the account?

This is not, like the other four questions, a yes/no question. It is a matter of estimating, in order of magnitude terms, the percentage change in fitness (= Survival times Reproduction) arising from the proposed use of language. So for the two leading accounts in Számadó and Szathmáry’s paper (hunting and tool-making), or for later accounts such as preparation for hunting (Számadó, 2010) or the confrontational scavenging scenario of (Bickerton and Szathmáry, 2011), we can try to estimate their percentage impact on the efficiency of food gathering, in the “survival” term of the product.

These percentage benefits can be estimated both for an early proto-language, and for more modern, highly capable fast language. In most scenarios, I would expect the additional benefit of modern fast language to be small, compared to the initial benefit of early language—because these uses of language typically do not require great speed of expression.

Sexual selection tends to be an all-or-nothing affair, of either passing on your genes, or failing to do so. Therefore it tends to lead to strong selection pressures—to a large percentage difference between “most attractive in the group” and “least attractive in the group.” Unlike the other scenarios, for sexual selection, the marginal benefits of faster, more powerful language over earlier slower language remain high throughout the whole evolution of language—because there is always competition to display more intelligence than your peers.

The overall evolution of language may have reflected both types of selection pressure (sexual and natural), so possibly a hybrid account could emerge. Particularly attractive is a serial hybrid model—in which ecological pressures such as the need for hunting or tool-making provided the initial impetus for the development of early language, as in (Számadó, 2010); and then later, sexual selection to display intelligence gave sustained selection pressure over a long period, to develop modern “supercharged” language. This model would solve two problems – providing a reason to kick-start sexual selection for intelligence, and a reason for modern uniquely human powerful language and intelligence.

Just as in physics, the strongest force determines the direction of motion, so in biology the strongest selection pressures determine the direction of evolutionary change. So at any period during the evolution of language, the dominant selection pressure would define the direction of change in language ability. Making such quantitative evaluations is a topic for future research.

Criteria for the comparative evaluation of different theories of the origin of language are also given by Botha (2016), who discusses “window phenomena” which bear indirectly on the evolution of language. His criteria for evaluating inferences from window phenomena are:

- The Empirical Requirement: Claims expressed in hypotheses about the evolution of language need to be empirical
- The Soundness Requirement: The inferential steps between whatever window phenomenon is said to illuminate the history of language evolution, and that history, must be sound steps. Inferences about language evolution need to meet specific soundness conditions, including warranting, pertinence and grounding.

Note that Botha’s grounding condition (about an account of language evolution) is not the same as Számadó and Szathmáry’s groundedness condition (about a use of early language being grounded in speaking about concrete things).

Botha uses his windows methodology to critique various lines of inference which proceed from physical or present-day window phenomena (such as markings and wear on marine shells in the Blombos caves from 75,000 years ago, or features of fossil cranial casts, or pidgins and creoles, or language acquisition) to conclusions about human language evolution at some time in the past—usually about its syntactic complexity. By these arguments he generally deconstructs these lines of inference, showing that one or more of their steps are unsound; and his arguments are persuasive. Botha’s conclusion is a pessimistic one, albeit a useful one; of the nine window inferences he examines, all are unsound—some of them in several ways. In his epilogue chapter, he does not pick out any of the nine window inferences as sound.

To apply all of Botha’s criteria to all the inferences in this paper would take up too much space. Nevertheless, I will illustrate the issues involved. In the account of language evolution by sexual selection, there are three main sources of empirical evidence:

- The progressive enlargement of the human brain, over more than 2 million years
- The prodigious speed and power of modern human language
- The uniqueness of human language and intelligence, compared to other great apes

These are well-documented phenomena, satisfying Botha’s empirical requirement. The bridging theories between these phenomena and the evolution of language are the theories of natural and sexual selection—which are well grounded and warranted, both empirically and theoretically.

The inferences which link the window phenomena to the evolution of language, *via* these bridging theories, form the body of this paper and cannot all be re-stated without lengthy repetition. I give one example:

- Human language and intelligence are uniquely powerful, unmatched by other great apes (empirical, grounded).
- Sexual selection often acts to give species-unique traits (empirical, grounded).
- There are theoretical reasons to expect sexual selection to lead to species-unique traits (inference, warranted).
- Therefore, sexual selection may account for the uniqueness of human language and intelligence.

This is a short chain of inference based on grounded facts and warranted inferences; and it accounts for a fact (human uniqueness) that other theories of language evolution do not account for Számadó and Szathmáry (2006). This inference satisfies Botha’s criteria. The account of language evolution by sexual selection is not an exclusive one; it is compatible with some other accounts, as a hybrid account. Therefore, to rebut the sexual selection account would require proving a negative—that sexual selection has never been involved in the evolution of human language, or cannot ever be involved. If that were to be asserted, I would welcome the discussion, in future publications.

## Other accounts of human brain enlargement

The early history of the hominin line is a complex story of several different speciations over several million years, built on fragmentary and partial fossil evidence, with large gaps. The story becomes more complex with each new fossil discovery. In these circumstances, even for questions such as brain size (for which there is direct fossil evidence) it can be hard to agree a progression [for instance, was brain enlargement steadily progressive, or did it proceed by plateaux and spurts, as advocated by Lynch and Granger (2008)]. For more abstract issues, where the evidence from fossils is at best indirect, it is even harder to agree on the progression or the evolutionary drivers (as is amply demonstrated in Botha (2016)). So it is not surprising if no single account is found to be satisfactory by all researchers.

A virtue of the account of human brain enlargement by sexual selection for the display of greater intelligence is that it proposes a driver of evolutionary change, which is not claimed to be the sole driver; and it does not claim to predict actual changes resulting from those drivers to a fine level of detail. Therefore the account from sexual selection is not an exclusive account, and may be compatible with many other accounts.

Nevertheless, there are accounts of human brain enlargement which appear to conflict with this account in various ways, and it is worth examining some of the potential conflicts.

Finlay and Darlington (1995) and Finlay et al. (2001) have analysed the sizes of the major sub-divisions of brains across a wide range of (131) mammalian species, and have found important regularities. They found that across a very wide range of mammalian brain sizes, the proportions of brain size in major brain regions (midbrain, diencephalon and cortex; and going down to 10 sub-structures within them) vary with total brain size in regular ways. These regularities apply to human brains, in spite of the fact that human brains are greatly enlarged compared to human body weights—well above the normal primate trend.

These regularities in the proportions of brain mass are remarkable, and are indicative of some underlying regularities in the development of mammalian brains; they may point to important developmental and genetic constraints. The regularities apply across a large number of mammalian species, many of which have been subject to sexual selection. Much sexual selection in animals includes selection in the cognitive traits of mate preference; so the regularities of Finlay & Darlington are consistent with sexual selection for cognitive traits—including superior intelligence. There is no *prima facie* inconsistency between their data and sexual selection.

Specifically, the regularities do not conflict with the account of human brain enlargement by sexual selection. In order to display superior intelligence by language, you first of all have to have superior intelligence—and this requires a larger brain, with no particular bias towards one region of the brain or another; so it is consistent with the data from Finlay & Darlington. The special extra requirement to display that intelligence through language may be modest compared with the requirement to have the extra intelligence in the first place. The special brain requirements for language may be no more demanding than the special requirements of other mammals (such as echo-location in bats) which are also met within the limits found by Finlay & Davidson. Cortical neural plasticity allows for great flexibility of function, consistent with the observed masses of major brain regions.

The regularities found by Finlay & Darlington have been used by Lynch and Granger (2008) to support a different picture of human brain enlargement. In chapter 11 of their book, they say that “*the big brain arose from the big baby, and the big baby arose first from changes in walking, and then in enlarged hips in females*.” This contradicts the assumption that selection pressures for intelligence drove the evolution of big brains; rather, they say, walking expanded brain size, and then we found uses for our greater intelligence.

Lynch & Granger draw support for their account from Finlay & Darlington, saying that “*the brain does not grow mosaically, differentially adapting itself to external pressures. It grows in uniform, concerted fashion, according to its own internal rules; and whatever behavioural rules happen to emerge from it are side-effects*.” However, the sexual selection pressure to display greater intelligence is not a differential, mosaic pressure; the need can be met by the brain growing in “*uniform concerted fashion, according to its own internal rules*;” so there is no contradiction.

The “big babies and walking” account of Lynch and Granger is an alternative to the account by sexual selection; but it appears to have a fundamental flaw. A larger brain has increased metabolic costs, which, by leading to greater food requirements, cause a decrease in fitness. Evolutionary change is always in the direction of increased fitness.

Lynch & Granger do not propose any reason why larger brains (in humans compared to other primates) should give greater fitness to compensate for their costs, and so to drive evolutionary change. They say that the metabolic costs of large brains can be compensated for by thermal regulation which is pervasive in mammals and birds, but not in reptiles; but this point is not relevant to the evolutionary change from apes to humans. If behavioural changes are only later side-effects, they cannot drive evolution.

Much of Lynch and Granger’s book is an account of the extra-large brains of Neanderthals and Boskops, which were larger than modern human brains. The sexual selection account of brain enlargement is a high-level view of a period of 2 million years; it does not give a detailed account of the last 300,000 year period within it (except for the last 10,000 years). So evidence about Neanderthals and Boskops does not affect the account, and may be consistent with sexual selection for intelligence. In particular, sexual selection could account for the fact that Neanderthals and Boskops are now extinct; their larger brains gave them individual benefits in reproductive fitness, but through their larger metabolic requirements, could have had a negative impact on their survival.

It has been suggested (Botha, 2020) that while Neanderthals had extra-large brains, they had only primitive language or no language. If Neanderthals had no language, that implies that the common ancestor of Neanderthals and *homo sapiens* had no language. This pushes the date of evolution of the language capacity to very late in the 2-million year period addressed this paper; so it effectively decouples the evolution of language from the prolonged enlargement of the human brain. This would remove an attraction of the sexual selection account, in linking the two; nevertheless, the late emergence of language remains a possible account to be tested.

No doubt there will continue to be many hypotheses about the causes of human brain enlargement, and of language; but at present, the account from sexual selection has some benefits, in linking the two, and in its account of the origin of our “supercharged,” uniquely human, language.

## Discussion

This paper has proposed that the capacity for language evolved in part by sexual selection for superior intelligence. This proposal is consistent with evidence that other accounts of the evolution of language do not account for:

- That language evolved in less than 2 million years
- That it allows us to communicate so fast, conveying complex messages within seconds—much faster than we need to for survival purposes
- That it has elaborate syntax and lexicon—more complex than is needed; for practical needs in a natural habitat, we could convey meanings more slowly
- That it constitutes an evolutionary discontinuity between mankind and the great apes
- That it comes with handicaps—particularly, the need for a large brain, with large food requirements

These are the hallmarks of sexual selection. They are consistent with fast complex language as a competitive sexual asset, attractive to both sexes, and used for the display of intelligence—as it is today. Because these points follow only from an account of language evolution by sexual selection, not from natural selection, it seems very likely that sexual selection has been involved in the evolution of language, at least in its later stages.

An account of language evolution through sexual selection is not incompatible with other accounts. Particularly attractive is a serial hybrid account, in which habitat needs such as hunting or tool-making provided the initial impetus for early language, and then later sexual selection gave sustained selection pressure towards powerful modern language.

The account hinges on the fact that sexual selection pressure, when it occurs, is stronger than natural selection pressures. Natural selection typically involves incremental changes, giving small increases in fitness as the habitat changes; whereas sexual selection is a winner-take-all competition to pass on your genes. This stronger sexual selection pressure has driven the evolution of the capacity for language—particularly in the later stages, leading to modern powerful language.

If language is seen as a means to display superior intelligence, it shifts the priorities in language research, away from the syntax-first approaches which have been dominant, to a pragmatics-first approach centred on iterative exchanges of knowledge. This relates language much more closely to other cognitive skills, as it is in cognitive linguistics. In this view, pragmatics is not just a dustbin for awkward phenomena left out of syntax and semantics; it is the beginning of language. A pragmatics-first approach mirrors the order in which young children learn language.

## Data availability statement

The original contributions presented in the study are included in the article/supplementary material, further inquiries can be directed to the corresponding author.

## Author contributions

The author confirms being the sole contributor of this work and has approved it for publication.

## Conflict of interest

The author declares that the research was conducted in the absence of any commercial or financial relationships that could be construed as a potential conflict of interest.

## Publisher’s note

All claims expressed in this article are solely those of the authors and do not necessarily represent those of their affiliated organizations, or those of the publisher, the editors and the reviewers. Any product that may be evaluated in this article, or claim that may be made by its manufacturer, is not guaranteed or endorsed by the publisher.
